# An empirical comparison of the measurement properties of the EQ-5D-5L, DEMQOL-U and DEMQOL-Proxy-U for older people in residential care

**DOI:** 10.1007/s11136-017-1777-0

**Published:** 2018-01-05

**Authors:** Tiffany Easton, Rachel Milte, Maria Crotty, Julie Ratcliffe

**Affiliations:** 1 Flinders Health Economics Group, College of Medicine and Public Health, Flinders University, Adelaide, SA Australia; 2 Institute for Choice, Business School, University of South Australia, Adelaide, SA Australia; 3 NHMRC Partnership Centre on Dealing with Cognitive and Related Functional Decline in Older People, University of Sydney, Sydney, NSW Australia; 4 Rehabilitation, Aged and Extended Care, College of Nursing and Health Sciences, Flinders University, Adelaide, SA Australia

**Keywords:** EQ-5D-5L, DEMQOL-U, Residential aged care, Quality of life, Dementia

## Abstract

**Purpose:**

This study aimed to empirically compare the measurement properties of self-reported and proxy-reported (in cases of severe cognitive impairment) generic (EQ-5D-5L) and condition-specific (DEMQOL-U and DEMQOL-Proxy-U) preference-based HRQoL instruments in residential care, where the population is characterised by older people with high rates of cognitive impairment, dementia and disability.

**Methods:**

Participants were recruited from seventeen residential care facilities across four Australian states. One hundred and forty-three participants self-completed the EQ-5D-5L and the DEMQOL-U while three hundred and eight-seven proxy completed (due to the presence of severe dementia) the EQ-5D-5L and DEMQOL-Proxy-U. The convergent validity of the outcome measures and known group validity relative to a series of clinical outcome measures were assessed.

**Results:**

Results satisfy convergent validity among the outcome measures. EQ-5D-5L and DEMQOL-U utilities were found to be significantly correlated with each other (*p* < 0.01) as were EQ-5D-5L and DEMQOL-Proxy-U utilities (*p* < 0.01). Both self-reported and proxy-reported EQ-5D-5L utilities demonstrated strong known group validity in relation to clinically recognised thresholds of cognition and physical functioning, while in contrast neither DEMQOL-U nor DEMQOL-Proxy-U demonstrated this association.

**Conclusions:**

The findings suggest that the EQ-5D-5L, DEMQOL-U and DEMQOL-Proxy-U capture distinct aspects of HRQoL for this population. The measurement and valuation of HRQoL form an essential component of economic evaluation in residential care. However, high levels of cognitive impairment may preclude self-completion for a majority. Further research is needed to determine cognition thresholds beyond which an individual is unable to reliably self-report their own health-related quality of life.

## Introduction

There is ongoing debate surrounding the ability of people with cognitive impairment and dementia to self-report their own health-related quality of life (HRQoL) [1, 2]. This issue is of particular importance for quality of life assessment and economic evaluations conducted in a residential care setting, where the majority of residents are living with cognitive impairment and dementia [3–6]. Presently, over 50% of residents in Australian residential aged care facilities have a diagnosis of dementia and estimates indicate that up to 75% are living with some form of cognitive impairment [3].

The EQ-5D is a generic preference-based instrument for the measurement and valuation of HRQoL that is widely applied in economic evaluation. It is well known for its reliability, responsiveness and validity in adults with good cognitive functioning [7]. The acceptability and feasibility of the EQ-5D for administration with individuals exhibiting mild to moderate cognitive impairment and living in residential aged care have been demonstrated in a number of published studies internationally [8–13]. For individuals who are unable to self-complete, the EQ-5D due to more severe levels of cognitive impairment, proxy assessment is necessary. A recent systematic review found proxy assessment of HRQoL to be most acceptable across the entire range of Alzheimer’s disease severity in terms of validity and reliability in detecting long-term changes relevant to economic evaluations [14]. The use of proxy assessors raises the question of who is the most appropriate proxy. Several studies have indicated that the choice of proxy respondent may also affect assessment of quality of life [13, 15]. A recent study undertaken in Spain by Diaz-Redondo and colleagues found that quality of life as measured by the EQ-5D was rated higher by staff members caring for people with dementia compared to ratings given by family members [16].

The DEMQOL and DEMQOL-Proxy were developed as condition-specific instruments to capture the measurement of HRQoL of individuals with cognitive decline and dementia where the DEMQOL was designed to be self-completed by the individual and the DEMQOL-Proxy was designed to be completed by a suitable proxy, e.g. close family member or a carer [17, 18]. In their original form, both the DEMQOL and the DEMQOL-Proxy are not suitable for use in economic evaluation as they provide summary scores that are not preference based. However, the recent development of the DEMQOL-U and DEMQOL-Proxy-U using general population preference values has facilitated the application of these measures in economic evaluations [19, 20].

A number of adjustments to the original EQ-5D instrument have emerged in recent years in a bid to improve various aspects of the instrument. An extended version of the EQ-5D was developed to incorporate a cognitive dimension in an effort to improve content validity [8]. This cognitive “bolt-on” has not yet been explored in depth, and it has not been incorporated into the scoring algorithm for the EQ-5D [9]. The recent development of a five-level version of the EQ-5D has improved its sensitivity compared to the original three-level version with an increase from 243 to 3125 unique health states [10]. To date, the five-level version of the EQ-5D has not been widely applied in residential care settings where the population is characterised by high rates of cognitive impairment, dementia and disability [11].

The main aim of this study was to empirically compare the measurement properties of the new five-level version of the EQ-5D (EQ-5D-5L), a generic preference-based measure of HRQoL with the DEMQOL-U and DEMQOL-Proxy-U condition-specific preference-based measures for older people living in residential care with cognitive impairment and dementia. Specifically, we assessed the convergent validity of the outcome measures relative to each other and relative to a battery of validated clinical outcome measures to assess cognition, functioning and neuropsychiatric symptoms for the self-reported and proxy-reported subgroups. We also assessed known group validity, a form of construct validity, by comparing the utilities derived from each instrument relative to the recognised severity thresholds relating to cognition and physical functioning.

## Methods

### Study participants

The data utilised for this study were derived from the INSPIRED (Investigating services provided in the residential care environment for dementia) study. The INSPIRED study was a cross-sectional, observational study to evaluate the specialised dementia services provided at residential aged care facilities in Australia. Data were collected from 17 residential aged care facilities across 4 states over a 14-month time period from January 2015 to February 2016. Residents were eligible to participate in this study if they (1) were permanent residents of the facility; (2) had been residing in the facility for at least 12 months; (3) were not in immediate palliative care; (4) had no complex medical or family issues which would impede their participation; and (5) had a family member willing to participate on their behalf if the resident themselves was unable to provide informed consent due to the presence of significant cognitive impairment.

### Data collection

The INSPIRED study involved the administration of a comprehensive set of outcome measurements, including generic and condition-specific measurements of health-related quality of life (assessed by the resident or proxy), cognitive function, dementia severity, physical function and neuropsychiatric symptoms (assessed by a care staff nurse).

### PAS-Cog

The Psychogeriatric Assessment Scales (PAS) are a collection of 6 scales which provide an assessment of dementia and depression in older adults [21]. The Cognitive Impairment scale consists of 9 questions administered in the form of an interview to test the cognitive functioning and memory of the subject. The resulting score ranges between 0 and 21, with 0 indicating that no impairment was detected by the scale and 21 indicating severe cognitive impairment.

### EQ-5D-5L

The EQ-5D-5L is a generic, preference-based instrument for the measurement and valuation of HRQoL [11]. The EQ-5D-5L can be completed by the subject or by a proxy, and collects subjective assessments of mobility, self-care, usual activities, pain/discomfort and anxiety/depression. Self-completion of the EQ-5D-5L was encouraged for all participating residents with a PAS-Cog score ≤ 11, based on evidence of its appropriateness in people with mild to moderate dementia [13, 22]. Health state utility values were generated from a scoring algorithm based on the time trade off (TTO) and discrete choice experiment (DCE) approaches in a UK general population sample [23]. Utility scores are bounded from − 0.281 to 1 where health states with a score less than 0 are considered worse than death. We also included a cognition bolt-on question which was originally developed for the 3-level version of the EQ-5D instrument [8]. The individual responses to the bolt-on question were not incorporated into the utility scoring algorithm as currently there is no recommended approach for facilitating this.

### DEMQOL-U and DEMQOL-Proxy-U

The DEMQOL and DEMQOL-Proxy are health-related quality of life instruments designed specifically for use in people with dementia [18]. The DEMQOL is a self-report instrument containing 28 items and is appropriate for use in people with mild to moderate dementia. Individual responses to the DEMQOL were converted to health state utility values using the DEMQOL-U scoring algorithm derived using the TTO approach in a UK general population sample [19]. The DEMQOL-U consists of four levels of severity in five dimensions: positive emotion, memory, relationships, negative emotion and loneliness. Utility scores for the DEMQOL-U are bounded from 0.243 to 0.986 [19]. The DEMQOL-Proxy, which contains 31 items, is designed for completion by a family member or carer. The DEMQOL-Proxy instructs proxies to provide responses to the instrument that most closely approximate the responses that they think the resident would provide themselves were they cognitively able to do so (proxy-resident perspective). Responses to the DEMQOL-Proxy were converted to health state utility values using the DEMQOL-Proxy-U scoring algorithm derived using the TTO approach in a UK general population sample [19]. The DEMQOL-Proxy-U consists of four levels of severity in four dimensions: positive emotion, memory, appearance and negative emotion. Utility scores for the DEMQOL-Proxy-U are bounded from 0.363 to 0.937 [19].

### Modified Barthel Index (MBI)

The MBI is a functional assessment scale which measures a person’s level of independence across a range of activities of daily living (ADL) functions [24]. Consisting of 10 items, the MBI is scored on a range from 0 to 100, with 0 indicating full dependence in all categories and 100 indicating full independence.

### Neuropsychiatric inventory questionnaire (NPI-Q)

The NPI-Q, a brief version of the original Neuropsychiatric Inventory, is a validated instrument for assessing psychopathology in dementia [25]. There is both a 10-item and 12-item version. The 10-item NPI-Q was completed by care staff for each participating resident in the INSPIRED study. Each of the items was rated by the care staff as 0–3 points according to levels of increasing severity with 0 indicating no symptoms were present in the past month, and 3 indicating severe symptoms were present.

### Recruitment process

The Psychogeriatric Assessment Scales–Cognitive Impairment Scale (PAS-Cog) was used to ascertain an eligible resident’s level of cognitive impairment [21]. PAS-Cog scores were collected from facility records if the assessment had occurred within 3 months of data collection and/or the individual had severe cognitive impairment (PAS-Cog ≥ 18). For all other participants, the PAS-Cog was administered by trained data collection personnel. Using PAS-Cog scores and/or advice from facility care staff, residents were separated into two separate consent profiles based on their likely ability to give informed consent and an appropriate recruitment approach was undertaken for each group. Residents who scored between 0 and 9 on the PAS-Cog (indicating no to mild cognitive impairment) were provided with information on the full INSPIRED study, and given time to consider whether they wished to participate. Proxy consent from a family member was sought for all eligible residents with moderate to severe cognitive impairment, or where the researcher had doubt regarding a resident’s ability to self-consent. Family members were initially sent the study information pack via post and then approached by telephone to determine whether they were interested in participating on behalf of the resident. Proxy consent was sought and completed by a family member, spouse or friend empowered with legal decision-making authority.

All outcome measures were collected via face-to-face interviews where possible. Where proxy consent was obtained, the proxy was invited to the aged care facility for a personal interview. If a personal interview was not possible, questionnaire packs were sent to the proxy via post with an option to either complete the questionnaires via a telephone interview or in their own time from the perspective of the resident.

### Statistical analysis

Summary statistics for the total INSPIRED study sample and the sub-samples of participants who self-completed and proxy completed the HRQOL instruments (EQ-5D-5L, DEMQOL-U and DEMQOL-Proxy-U) were calculated for all demographic and outcome measures. The distribution of each variable was assessed for normality using the Kolmogorov–Smirnov test with Lilliefors significance correction and the Shapiro–Wilks test. Utility distributions were plotted for the EQ-5D-5L, DEMQOL-U and DEMQOL-Proxy-U. The Mann–Whitney *U* test and the Kruskal–Wallis test were used to compare utility scores across subgroups.

The strength of association between the dimensions of the EQ-5D-5L, DEMQOL-U and DEMQOL-Proxy-U were evaluated using Spearman’s rank order correlations and Index-level correlations were graphically represented with scatterplots. Correlation sizes below 0.3 were considered weak, those from 0.3 to < 0.5 were considered moderate, and those from 0.5 to < 0.6 were considered strong, and those of 0.6 or greater were considered very strong [19, 26]. The level of agreement between the HRQoL instruments was also graphically presented using Bland–Altman plots [27]. Differences in individual-level utility scores were plotted on the *y* axis, and average utility scores were plotted on the *x* axis. For each Bland–Altman plot, a mean difference was calculated along with 95% limits of agreement (LOA), equal to the mean difference ± 1.96 standard deviations of the difference. A mean difference value that differed significantly from 0 indicated the presence of a fixed bias. The limits of agreement indicated how far apart measurements by each instrument were likely to be for an individual.

Convergent validity for the EQ-5D-5L and the DEMQOL-U was assessed by examining the mean distributions of DEMQOL-U utilities by EQ-5D-5L dimensions. Convergent validity was also assessed between each HRQoL instrument and three common clinical outcome measures for dementia: cognitive function, physical function and neuropsychiatric symptoms. Bivariate correlation coefficients were calculated for each HRQoL dimension with the respective clinical outcome measures (PAS-Cog, MBI, and NPI-Q). Known- group validity was assessed using clinically severity thresholds PAS-Cog and MBI. Specifically, we hypothesised that participants with no to mild cognitive impairment (PAS-Cog score 0–9) would have higher utilities relative to those with moderate (PAS-Cog score 9–15) and severe (PAS-Cog score 16–21) impairment. Similarly, we hypothesised that participants with better physical functioning as measured by the MBI would have higher utilities than those with worse physical functioning. Effect size indices were also calculated to provide an indication of the average group difference taking into account the variability observed in the group with least impairment (PAS-Cog score 0–3; MBI score = 100). Based on commonly cited guidelines, values of 0.2, 0.5 and 0.8 denote small, medium and large effect sizes, respectively [28].

## Results

The INSPIRED study assessed a total of 1323 people living in 17 residential care facilities across 4 Australian states. The facilities belonged to 5 not-for-profit aged care organisations. Of the total resident pool, 901 met eligibility requirements and 541 were consented to the study. A total of 24% of study participants self-consented, while proxy consents were obtained for 76%. The mean (SD) age of participants was 85.5 (8.5) years. Descriptive statistics for the INSPIRED study sample as well as the subgroups who self-reported and proxy-reported HRQOL are presented in Table 1.

**Table 1:** Sample characteristics and summary statistics for the INSPIRED study sample and self-rated HRQoL subgroup

Variable	INSPIRED study sample			Self-rated HRQoL subgroup			Proxy-rated HRQoL subgroup		
	*N*	Mean (SD) or %	Range	*N*	Mean (SD) or %	Range	*N*	Mean (SD) or %	Range
Participant characteristics									
Age (years)	541	85.5 (8.5)	48–104	143	85.7 (8.8)	49–99	387	85.5 (8.3)	48–104
Female	403	74. 5	–	103	72.0	–	290	74.9	–
Diagnosis of dementia	345	64.0	–	35	24.5	–	299	77.3	–
Self-rated HRQoL utility values									
EQ-5D-5L	151	0.66 (0.29)	– 0.28 to 1.00	143	0.66 (0.28)	– 0.28 to 1.00	–	–	–
DEMQOL-U	225	0.85 (0.12)	0.30–0.99	143	0.87 (0.12)	0.39–0.99	–	–	–
Proxy-rated HRQoL utility values									
EQ-5D-5L-Proxy	390	0.48 (0.29)	– 0.28 to 1.00	–	–	–	387	0.48 (0.29)	– 0.28 to 1.00
DEMQOL-Proxy-U	536	0.69 (0.13)	0.36–0.94	–	–	–	387	0.68 (0.13)	0.36–0.94
Clinical outcome measures									
PAS-Cog score (max 21)	520	13.2 (7.7)	0–21	143	3.75 (2.8)	0–11	366	16.6 (5.9)	0–21
Modified Barthel Index (max 100)	537	40.2 (32.7)	0–100	143	63.0 (30.0)	0–100	383	32.9 (29.8)	0–100
NPI-Q 10 item sum severity (max 30)	538	8.3 (6.4)	0–28	143	4.9 (4.7)	0–25	384	9.5 (6.5)	0–28

In the full INSPIRED study sample (*n* = 541), a measurable level of cognitive impairment was present in 83% of participants and 64% had a recorded diagnosis of dementia. Among the subset of 143 participants who were able to complete both the DEMQOL-U and EQ-5D-5L instruments, 45% were identified as living with mild or moderate cognitive impairment, and 25% had a diagnosis of dementia. Among the sub-sample of 387 participants for which both the DEMQOL-Proxy-U and EQ-5D-5L instruments were completed by proxy, 77% had a recorded diagnosis of dementia. Twenty-one participants in the proxy-rated subgroup were unable to complete the PAS-Cog due to severe cognitive impairment.

**Fig. 1:**
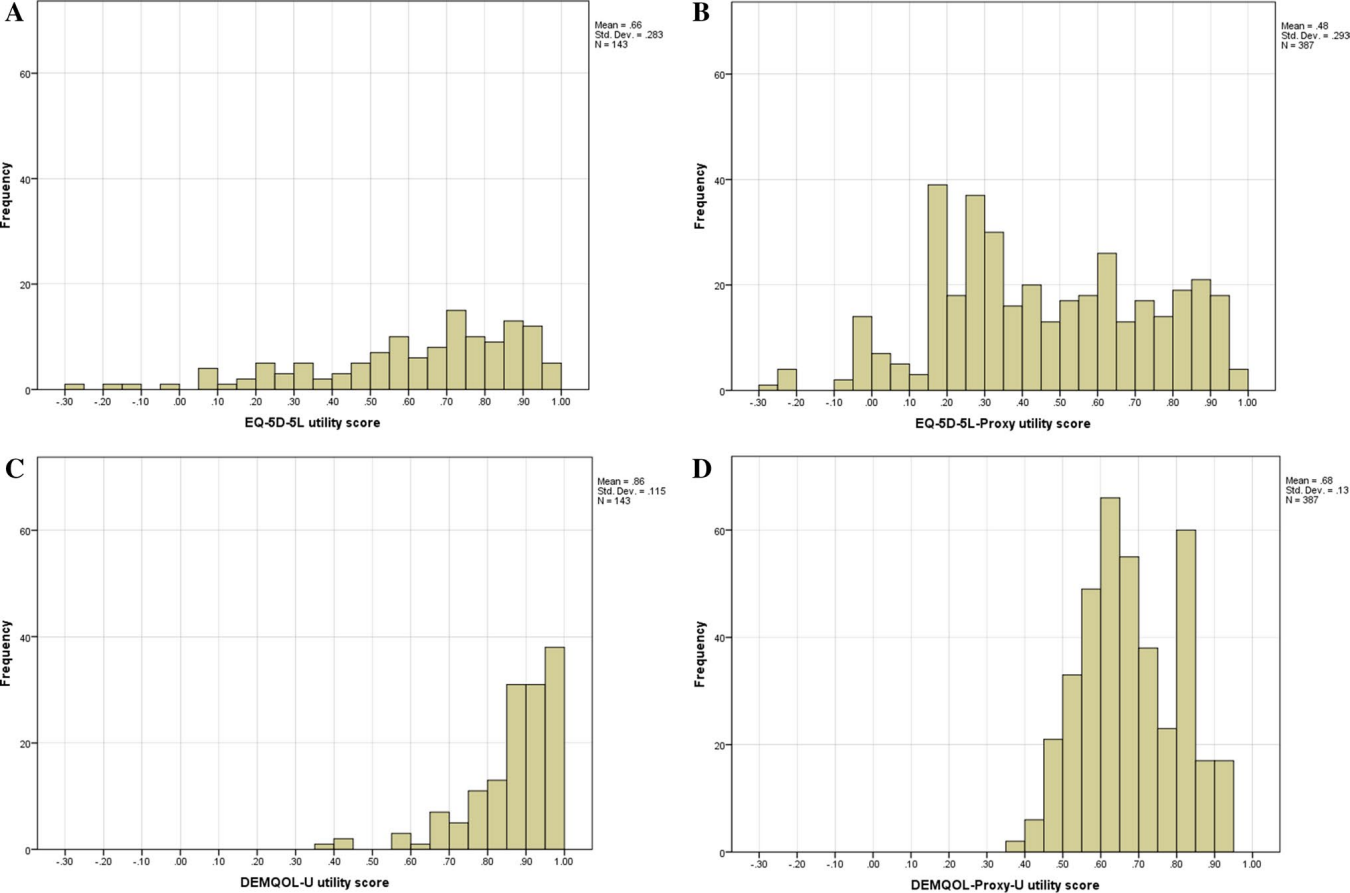
Distribution of EQ-5D-5L and DEMQOL-U, EQ-5D-5L-Proxy and DEMQOL-Proxy-U utility scores

For the self-completion sub-sample neither the EQ-5D-5L nor DEMQOL-U instruments produced normally distributed values according to the Kolmogorov–Smirnov test with Lilliefors significance correction and the Shapiro–Wilks test. A negative skew was observed for both instruments (Fig. 1). No significant differences in utility scores were found between males and females for either instrument. EQ-5D-5L utility values tended to increase with age (*p* = 0.033), with the older residents reporting higher utility scores on average than younger residents. This age-related trend was not found in DEMQOL-U scores. Mean EQ-5D-5L utility scores were higher for residents with a diagnosis of dementia compared to those without diagnosed dementia and these differences were found to be statistically significant (*p* = 0.001). This pattern was also evident for the DEMQOL-U, with slightly higher scores reported on average for those with a diagnosis of dementia, however, this difference was not found to be statistically significant (*p* = 0.105).

For the proxy-completed sub-sample, similarly neither the EQ-5D-5L nor DEMQOL-Proxy-U instruments produced normally distributed values according to the Kolmogorov–Smirnov test with Lilliefors significance correction and the Shapiro–Wilks test. The distributions of utility scores for the completed by proxy are shown in Fig. 1. No significant differences in utility scores were found between males and females for either instrument. No associations were found between utility scores and resident age for either instrument. Mean EQ-5D-5L proxy utility scores were slightly higher for residents with a diagnosis of dementia compared to those without diagnosed dementia; however, the difference was not statistically significant (*p* = 0.314). Mean DEMQOL-Proxy-U utility scores were the same for residents with and without a diagnosis of dementia.

**Fig. 2:**
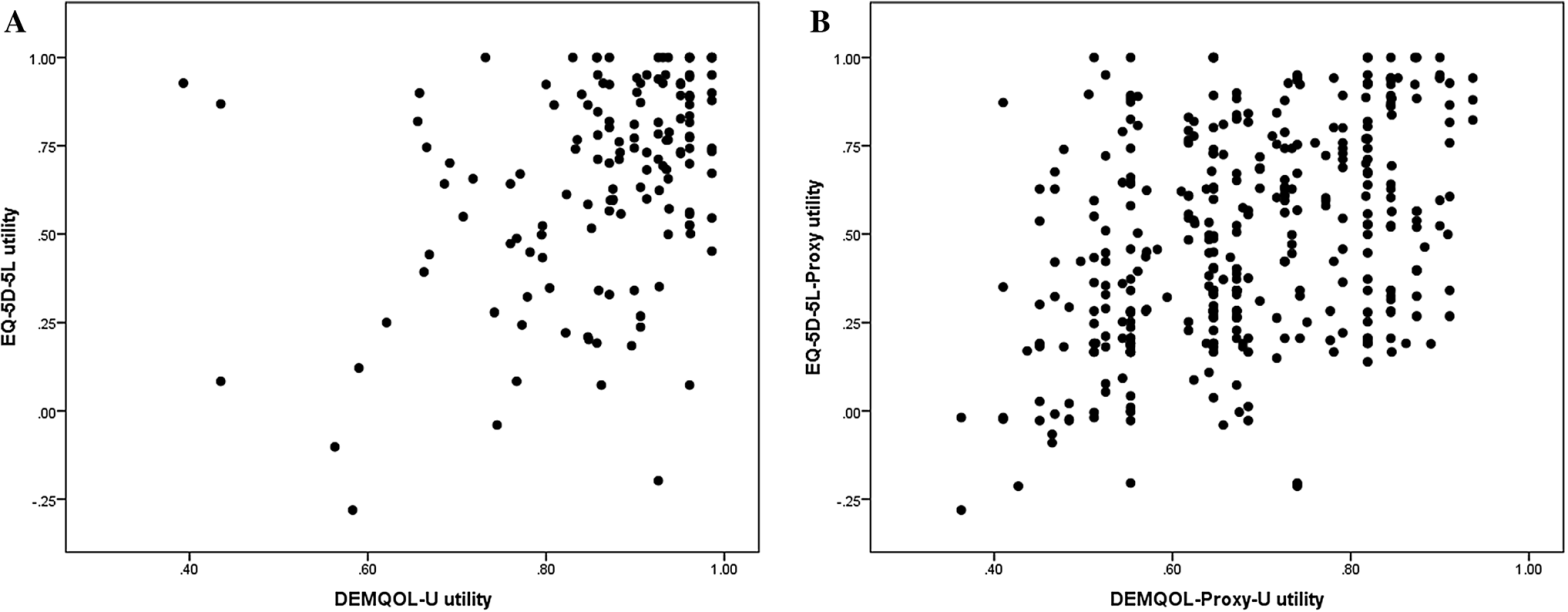
Scatterplot of EQ-5D-5L and DEMQOL-U, and EQ-5D-5L-Proxy and DEMQOL-Proxy-U utility values

The Spearman correlation coefficients between the EQ-5D-5L and DEMQOL-U and EQ-5D-5L and DEMQOL-Proxy-U, respectively, are presented in Table 2, and graphically in Fig. 2. Generally speaking, for both sub-samples, the correlations were weak to moderate across all dimensions. Eleven participants described themselves in full health according to the EQ-5D-5L but also with at least some impairment in the DEMQOL-U. Similarly, eleven participants were described in full health according to the EQ-5D-5L by proxy but also with at least some impairment in the DEMQOL-Proxy-U. In contrast, 8 participants described themselves in full health according to the DEMQOL-U while their corresponding EQ-5D-5L scores indicated a range of impairments and 3 participants were described in full health according the DEMQOL-Proxy-U while their corresponding EQ-5D-5L scores also indicated a range of impairments.

**Table 2:** Spearman correlation coefficients of EQ-5D-5L, DEMQOL-U, and DEMQOL-Proxy-U measures

Self-reported HRQoL measures						
	Positive emotion	Negative emotion	Loneliness	Cognition	Relationships	DEMQOL-U Index
Mobility	0.150	− 0.059	− 0.048	− 0.154	− 0.215^**^	− 0.134
Self-care	0.251^**^	− 0.157	− 0.181^*^	− 0.125	− 0.191^*^	− 0.267^**^
Usual activities	0.163	− 0.260^**^	− 0.262^**^	− 0.156	− 0.324^**^	− 0.351^**^
Pain and discomfort	0.151	− 0.203^*^	− 0.215^**^	− 0.220^**^	− 0.194^*^	− 0.294^**^
Anxiety and depression	0.278^**^	− 0.371^**^	− 0.204^*^	− 0.253^**^	− 0.177^*^	− 0.374^**^
Cognition	0.181^*^	− 0.158	− 0.120	− 0.304^**^	− 0.234^**^	− 0.222^**^
EQ-5D-5L Index	− 0.231^**^	0.255^**^	0.216^**^	0.216^**^	0.321^**^	0.346^**^

Proxy-reported HRQoL measures						
	Negative emotion	Positive emotion	Memory	Appearance	DEMQOL-Proxy-U Index	
Mobility	− 0.106*	0.282**	0.049	0.021	− 0.265**	
Self-care	− 0.036	0.307**	0.058	0.055	− 0.248**	
Usual activities	− 0.077	0.344**	0.022	0.058	− 0.306**	
Pain and discomfort	− 0.338**	0.079	− 0.131*	− 0.136**	− 0.249**	
Anxiety and depression	− 0.381**	0.131**	− 0.232**	− 0.146**	− 0.334**	
Cognition	0.025	0.243**	− 0.005	0.128*	− 0.177**	
EQ-5D-5L Index	0.251**	-0.325**	0.049	0.027	0.389**	

*Correlation is significant at the 0.05 level (2-tailed)

**Correlation is significant at the 0.01 level (2-tailed)

**Fig. 3:**
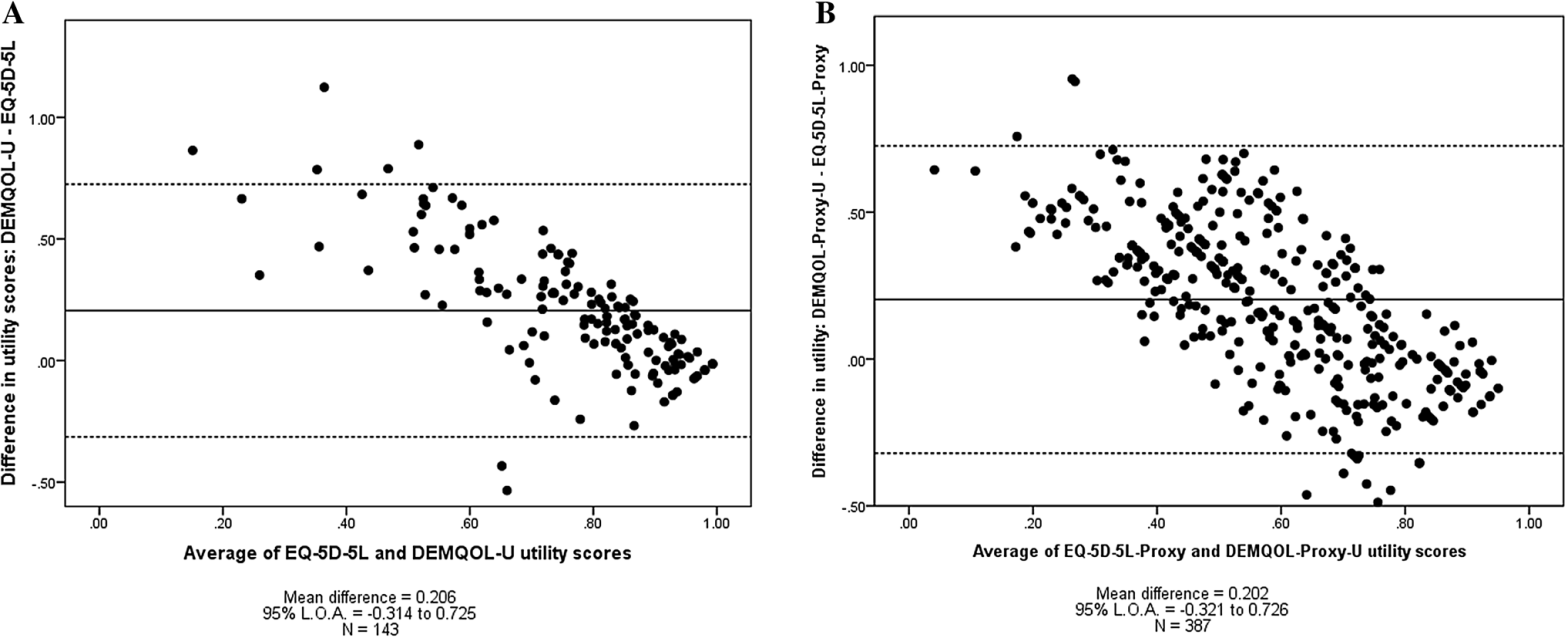
Bland–Altman plots analysing agreement between EQ-5D-5L and DEMQOL-U, and EQ-5D-5L-Proxy and DEMQOL-Proxy-U

The agreements between utility scores generated by the EQ-5D-5L and DEMQOL-U, and EQ-5D-5L and DEMQOL-Proxy-U, respectively, are graphically presented in Fig. 3. The mean difference for the self-reported instruments was 0.206, with the 95% LOA ranging from − 0.314 to 0.725. The mean difference for the proxy-rated instruments was 0.202, with the 95% LOA ranging from − 0.321 to 0.726. For both sub-samples, there is evidence of a higher level of agreement between instruments at higher levels of utility, with more responses clustering around the zero mean difference. At lower levels of utility both the DEMQOL-U and DEMQOL-Proxy-U produce consistently higher utility values than the EQ-5D-5L.

The correlations between the HRQoL dimensions and clinical outcome measures are presented in Table 3. For both sub-samples, overall, the correlations between the EQ-5D-5L and clinical outcome measures were stronger than between the DEMQOL-U/DEMQOL-Proxy-U and clinical outcome measures. In both sub-samples, physical functioning as measured by the MBI showed a moderately strong correlation with EQ-5D-5L utilities, particularly in the dimensions of “mobility”, “self-care” and “usual activities” with greater impairments being associated with lower reported quality of life. Higher levels of cognitive impairment, as measured by the PAS-Cog, were associated with higher self-reported quality of life as measured by the EQ-5D-5L, particularly in the dimensions of “mobility”, “self-care” and “pain and discomfort”, although the strength of the correlation (*r* = 0.24) was weak. Behavioural and psychological symptoms, as measured by the NPI-Q, were weakly correlated with the DEMQOL-U Index and the dimension of “loneliness” in particular with more severe symptoms associated with higher reported loneliness and lower quality of life overall. Behavioural and psychological symptoms, as measured by the NPI-Q, were weakly correlated with the DEMQOL-Proxy-U dimension of “negative emotion”. There was also a weak to negligible correlation between physical function and the “positive emotion” and “appearance” dimensions of the DEMQOL-Proxy-U with better function associated with higher positive emotion and appearance.

**Table 3:** Spearman correlation coefficients of EQ-5D-5L and DEMQOL-U measures with clinical outcome measures

	Cognition (PAS-Cog)	Physical function (MBI)	Behavioural and psychological symptoms (NPI-Q)
Self-reported HRQoL			
EQ-5D-5L			
Mobility	− 0.230^**^	− 0.499^**^	0.018
Self-care	− 0.237^**^	− 0.609^**^	0.069
Usual activities	− 0.087	− 0.374^**^	0.140
Pain and discomfort	− 0.194^*^	− 0.065	0.143
Anxiety and depression	− 0.004	− 0.050	0.076
Cognition	0.025	− 0.039	0.045
EQ-5D-5L Index	0.243^**^	0.492^**^	− 0.099
DEMQOL-U			
Positive emotion	− 0.047	− 0.183^*^	0.102
Negative emotion	0.011	0.119	− 0.116
Loneliness	0.028	0.010	− 0.201^*^
Cognition	− 0.100	0.003	− 0.043
Relationships	− 0.062	0.094	− 0.003
DEMQOL-U Index	0.066	0.105	− 0.183^*^
Proxy-reported HRQoL			
EQ-5D-5L-Proxy			
Mobility	0.215**	− 0.555**	− 0.177**
Self-care	0.386**	− 0.627**	− 0.063
Usual activities	0.356**	− 0.577**	− 0.083
Pain and discomfort	− 0.067	− 0.055	0.006
Anxiety and depression	− 0.043	0.000	0.160**
Cognition	0.495**	− 0.488**	0.062
EQ-5D-5L Index	− 0.261**	0.560**	0.056
DEMQOL-Proxy-U			
Negative emotion	0.023	− 0.058	− 0.231**
Positive emotion	0.108*	− 0.147**	− 0.051
Memory	0.124*	− 0.131*	− 0.069
Appearance	0.163**	− 0.133**	− 0.013
DEMQOL-Proxy-U Index	− 0.048	0.069	− 0.057

*Correlation is significant at the 0.05 level (2-tailed)

**Correlation is significant at the 0.01 level (2-tailed)

Table 4 indicates that no particularly strong relationships were evident between proxy-reported HRQoL utilities and cognitive impairment. There was a relationship between self-reported EQ-5D-5L and cognitive impairment although this was not in the expected direction. Participants with more severe cognitive impairment reported themselves with higher HRQoL than those with lower levels of cognitive impairment. This relationship was also evident for the DEMQOL-U but the differences in mean values were not as pronounced. However, these results should be interpreted with caution as the effect sizes are small and the n is particularly small for the moderate impairment group. Table 4 also indicates that EQ-5D-5L scores decrease across physical function severity groups in the expected direction for both the self-reported and proxy-completed sub-samples and the effect sizes are in the small to moderate range. The DEMQOL-Proxy-U scores also decreased across physical function severity groups in the expected direction but the differences were not as pronounced as for the EQ-5D-5L.

**Table 4:** Self-reported and Proxy-reported HRQoL utilities (mean and SD) by clinical determinants (PAS-Cog, and MBI)

Sample	Characteristic	*N*	EQ-5D-5L		DEMQOL-U/DEMQOL-Proxy-U	
			Mean (SD)	Effect size	Mean (SD)	Effect size
Self-Reported HRQoL	Cognitive impairment					
	No or minimal (PAS-Cog score 0–3)	78	0.61 (0.29)	–	0.86 (0.11)	–
	Mild (PAS-Cog score 4–9)	60	0.70 (0.27)	0.18	0.86 (0.12)	0.04
	Moderate (PAS-Cog score 10–15)	5	0.95 (0.08)	0.31	0.91 (0.06)	0.08
	Severe (PAS-Cog score 16–21)	0	–	–	–	–
	*p* value		0.002		0.667	
	Physical function					
	Independence (MBI score 100)	11	0.87 (0.12)	–	0.90 (0.08)	–
	Slight dependence (MBI score 91–99)	22	0.73 (0.22)	0.36	0.87 (0.09)	0.19
	Moderate dependence (MBI score 61–90)	52	0.74 (0.24)	0.05	0.87 (0.12)	0.12
	Severe dependence (MBI score 21–60)	38	0.61 (0.26)	0.29	0.84 (0.12)	0.20
	Total dependence (MBI score 0–20)	24	0.37 (0.32)	0.38	0.87 (0.13)	0.23
	*p* value		< 0.001		0.154	
Proxy-Reported HRQoL	Cognitive impairment					
	No or minimal (PAS-Cog score 0–3)	16	0.50 (0.34)	–	0.62 (0.09)	–
	Mild (PAS-Cog score 4–9)	43	0.60 (0.29)	0.11	0.71 (0.13)	0.27
	Moderate (PAS-Cog score 10–15)	64	0.53 (0.25)	0.15	0.69 (0.14)	0.08
	Severe (PAS-Cog score 16–21)	243	0.44 (0.30)	0.15	0.67 (0.13)	0.01
	*p* value		0.001		0.277	
	Physical function					
	Independence (MBI score 100)	2	0.84 (0.14)	–	0.83 (0.15)	–
	Slight dependence (MBI score 91–99)	14	0.73 (0.11)	0.24	0.75 (0.11)	0.28
	Moderate dependence (MBI score 61–90)	69	0.67 (0.23)	0.08	0.69 (0.13)	0.15
	Severe dependence (MBI score 21–60)	128	0.57 (0.27)	0.18	0.68 (0.13)	0.02
	Total dependence (MBI score 0–20)	169	0.31 (0.25)	0.47	0.67 (0.13)	0.04
	*p* value		< 0.001		0.134	

The Kruskal–Wallis test was used to calculate *p* values when testing for differences across known groups. The Mann–Whitney *U* test was then used to calculate effect size between groups. Effect size was calculated by dividing *Z* by the square root of *N*

## Discussion

This study presents one of the first empirical comparisons in Australia and internationally of the measurement properties of the EQ-5D-5L, DEMQOL-U and DEMQOL-Proxy-U instruments and represents the first study internationally to empirically compare the measurement properties of these instruments in a residential care setting. The central importance of proxy assessment of HRQoL in this setting is highlighted, with 75% of our study participants having insufficient cognitive ability to self-assess their own HRQoL. With the exception of the moderate correlation found between physical function and the EQ-5D-5L, the clinical outcome measures for people with dementia—cognition, physical function and neuropsychological symptoms—showed little association with the utility scores produced by the EQ-5D-5L and DEMQOL-U instruments. This is consistent with the findings of other studies conducted in older people living with cognitive impairment and/or dementia in community-based settings which have reported little or no association between clinical outcome measures for dementia and self-reported quality of life as measured by the EQ-5D-3L [19, 29–31] and DEMQOL-U [19].

Higher levels of cognitive impairment were associated with higher self-reported EQ-5D-5L utilities. Interestingly, overall mean utility scores for residents with a diagnosis of dementia were higher than for those without a diagnosis of dementia. It is possible that people admitted to residential care without a diagnosis of dementia have more severe physical disabilities than those with a dementia diagnosis and this could account for such differences. Previous research conducted in Australia has found people living in residential aged care facilities with a diagnosis of dementia tend to have higher care needs on average than those without in relation to activities of daily living and behaviour [3]. In our sample, however, the subgroup without dementia had a lower MBI score on average indicating a higher level of dependence, while the subgroup with dementia had a higher average NPI-Q sum score and higher average PAS-Cog score suggesting more cognitive impairment and more behavioural and psychological symptoms than residents without a dementia diagnosis. This finding was not repeated in the proxy-rated subgroup and these results should be interpreted with caution as the effect sizes were small. Proxy-rated utility values did not differ between residents with and without dementia. While this may seem counterintuitive, especially for a dementia-specific instrument like the DEMQOL-Proxy-U, it is consistent with the existing literature which has found no association between quality of life measures and cognitive function or dementia severity [32, 33]. The implication of this finding, as highlighted in Banerjee et al. [32], is that discrete measures such as cognition are likely to miss important factors which contribute to better quality of life in residential care.

In terms of the utility scores generated by the EQ-5D-5L and DEMQOL-U, the mean difference was 0.2 with utility scores generated by the EQ-5D-5L tending to be lower than those generated by the DEMQOL-U. The 95% LOA indicated that DEMQOL-U scores tended to range from 0.7 above their corresponding EQ-5D-5L score to 0.3 below. This apparent disagreement must be interpreted with caution given the different lower bound of each instrument. Despite both instruments providing a score on the theoretical 0 = dead − 1 = full health utility scale required for the calculation of quality adjusted life years (QALYS) the findings from this study indicate that the DEMQOL-U is not an appropriate substitute for the EQ-5D-5L and vice-versa.

Our data suggest that the DEMQOL-U and EQ-5D-5L capture distinct and unique aspects of HRQoL as indicated by poor agreement and low to negligible correlations across all dimensions. Ceiling effects were apparent for both the EQ-5D-5L and DEMQOL-U dimensions which is a commonly reported occurrence in self-reported measures for people with dementia [13, 19, 22]. The DEMQOL-U demonstrated a reasonable ability to discriminate between the dimensions of the EQ-5D-5L, with utility scores tending to decrease as EQ-5D-5L dimensions worsened. This pattern was much more consistent in the more psycho-social dimensions “pain and discomfort”, “anxiety and depression” and “cognition” compared to the more physical dimensions of “mobility”, “self-care” and “usual activities”. This may be explained by the psycho-social nature of the DEMQOL-U dimensions. In contrast, the EQ-5D-5L was found to be more strongly related to physical functioning as assessed by the MBI. The EQ-5D-5L may therefore be a more suitable instrument for the assessment of HRQoL in mixed residential care populations that include people with dementia but also people with co-morbidities, high levels of physical disability and frailty with good cognition.

Both instruments collected data on a “cognition” dimension, although the cognition dimension was not included in the Index-score calculation for the EQ-5D-5L. The cognition dimension had only a low positive correlation (*r* = 0.3, *p* < 0.01) between the two instruments. It is possible that this empirical difference is related to the differential framing of the question relating to cognition for each instrument. The EQ-5D-5L asks participants to answer to best describe how they are feeling today with the cognition dimension options ranging from “I do not have any problems with cognitive functioning” to “I have extreme problems with cognitive functioning”. In contrast, the DEMQOL asks participants “In the last week how worried have you been about forgetting things that happened recently?” with possible responses ranging from “a lot” to “not at all”.

It is important to note that the majority of previous studies reporting upon the HRQoL of people with dementia have predominantly been conducted in community based settings. Of the small number of recent studies investigating quality of life in a residential care setting, the vast majority have used proxy-rated instruments [16, 34] and/or mapping techniques [35] to elicit health state values for people with dementia. Similarly, a study measuring quality of life among hospital in-patients with dementia concluded that proxy-ratings were the only feasible option [36]. We have demonstrated that self-assessment of own HRQoL is feasible for at least a proportion of people living in residential care. Self-assessment of HRQoL by residents themselves is preferable where possible [7]. Given the results of our analyses, we are inclined to conclude that proxy-rated measures of HRQoL may be a practical option for the elicitation of health state values for use in cost–utility analyses in a residential aged care setting in order to ensure a consistently representative study sample and to facilitate longitudinal assessment of the HRQoL of all residents. Above and beyond practicality, however, there are important ethical considerations in deciding whether to use self or proxy measures. This study did not directly compare self and proxy versions of the same instrument, rather it compared generic with dementia-specific instruments for distinct self and proxy subgroups. The results of studies which directly compare self and proxy responses for the same participants should also be considered, along with factors such as proxy bias [37–39].

There are several limitations to our study. Participation at organisational level, facility level and individual level was voluntary and thus the participants in this study may not be representative of the Australian residential care population. The scoring algorithms for both the EQ-5D-5L and DEMQOL-U were from a UK general population sample despite the study having taken place in Australia. Further empirical analyses are warranted when Australian scoring algorithms become available, as utilities for identical health states have been shown to differ across countries and jurisdictions [40, 41]. It should also be noted that the UK EQ-5D-5L scoring algorithm has been revised since our analysis was completed [42]. Changes in the revised algorithm were minor and the Euroqol group notes that the practical implications of these changes are relatively small. We are therefore highly confident that this change will not affect the overall conclusions of our research. Future studies conducted in the Australian context should use the updated UK algorithm until the Australian EQ-5D-5L general population-specific scoring algorithm, currently in development, becomes publically available. Finally, as the study was cross-sectional in nature, it was not possible to assess either instrument’s responsiveness to change over time, an important criteria for the conduct of economic evaluation studies.

## Conclusions

The EQ-5D-5L, DEMQOL-U and DEMQOL-Proxy-U capture distinct aspects of HRQoL which provide useful complements to clinical outcome measures for people with dementia. Although these instruments were designed to measure the same concept of utility on an equivalent quality adjusted life years (QALY) scale, it is apparent that QALYs produced by the condition-specific DEMQOL-U are not directly comparable with QALYs produced by the EQ-5D-5L. Researchers and decision-makers should therefore be cautious in their interpretation of cost–utility analyses and pay careful attention to the outcome measures used in the assessment of effectiveness and the calculation of QALYs. With its strong association with physical functioning, the EQ-5D-5L may be a more suitable instrument for the assessment of HRQoL in mixed residential care populations that include people with dementia but also people with co-morbidities, high levels of physical disability and frailty with good cognition. The DEMQOL-U and DEMOL-Proxy-U, on the other hand, may be suitable for dementia-specific interventions that are more psycho-social in nature.

The measurement and valuation of HRQoL provide an important outcome which is highly relevant for quality assessment and economic evaluations conducted in residential care settings. Further research should be directed towards informing the highly important but vexed question as to the level of cognitive impairment beyond which an individual is unable to reliably self-report generic preference-based health-related quality of life instruments, including the EQ-5D, and beyond which proxy assessment should be sought.
